# Development and validation of a pain monitoring app for patients with musculoskeletal conditions (The Keele pain recorder feasibility study)

**DOI:** 10.1186/s12911-019-0741-z

**Published:** 2019-01-25

**Authors:** John Bedson, Jonathon Hill, David White, Ying Chen, Simon Wathall, Stephen Dent, Kendra Cooke, Danielle van der Windt

**Affiliations:** Arthritis Research UK Primary Care Centre, Research Institute for Primary Care & Health Sciences, Keele University, Keele, Staffordshire ST5 5BG UK

**Keywords:** Pain, Assessment, Musculoskeletal, Primary care, Smartphone, Application, App, eHealth, Telehealth

## Abstract

**Background:**

Assessing daily change in pain and related symptoms help in diagnosis, prognosis, and monitoring response to treatment. However, such changes are infrequently assessed, and usually reviewed weeks or months after the start of treatment. We therefore developed a smartphone application (Keele Pain Recorder) to record information on the severity and impact of pain on daily life. Specifically, the study goal was to assess face, content and construct validity of data collection using the Pain Recorder in primary care patients receiving new analgesic prescriptions for musculoskeletal pain, as well as to assess its acceptability and clinical utility.

**Methods:**

The app was developed with Keele’s Research User Group (RUG), a clinical advisory group (CAG) and software developer for use on Android devices. The app recorded pain levels, interference, sleep disturbance, analgesic use, mood and side effects. In a feasibility study, patients aged > 18 attending their general practitioner (GP) with a painful musculoskeletal condition were recruited to use the app twice per day for 28 days. Face and construct validity were assessed through baseline and post-study questionnaires (Spearman’s rank correlation coefficient). Usability and acceptability were determined through post-study questionnaires, and patient, GP, RUG and CAG interviews.

**Results:**

An app was developed which was liked by both patients and GPs. It was felt that it offered the opportunity for GPs to discuss pain control with their patients in a new way. All participants found the app easy to use (it did not interfere with their activities) and results easy to interpret. Strong associations existed between the first 3 days (Spearman *r* = 0.79) and last 3 days (*r* = 0.60) of pain levels and intensity scores on the app with the validated questionnaires.

**Conclusions:**

Collaborating with patient representatives and clinical stakeholders, we developed an app which can be used to help clinicians and patients monitor painful musculoskeletal conditions in response to analgesic prescribing. Recordings were accurate and valid, especially, for pain intensity ratings, and it was easy to use. Future work needs to examine how pain trajectories can help manage changes in a patient’s condition, ultimately assisting in self-management.

**Electronic supplementary material:**

The online version of this article (10.1186/s12911-019-0741-z) contains supplementary material, which is available to authorized users.

## Background

Annually, 15–20% of all adults attending general practice present with musculoskeletal conditions [1]. Around 40% of these patients are prescribed analgesia during their first consultation for musculoskeletal pain, half of whom will receive a non-steroidal anti-inflammatory drug (NSAID), and 29% a moderate to strong opioid containing analgesic [2, 3]. The general practitioner’s (GP’s) intention is to relieve their patient’s pain and the decision to use analgesia, for example in low back pain, is often based on the patient’s verbal report of their pain and their personal analgesic preferences [4]. Any subsequent review of analgesic efficacy is similarly limited by the GP relying on the patient’s reports of their pain progress, often quantified by asking patients to rate their pain on a scale from 0 (no pain) to 10 (worst pain) [5]. However, this existing approach to pain monitoring fails to account for the multiple dimensions of pain experience in relation to its onset (acute, or gradually developed over time), course over time (stable or highly variable), severity, and impact on everyday life [6]. Obtaining more detailed pain experience information could be an important and useful tool for clinical practice. For example, symptom trajectories have been shown to help obtain an accurate diagnosis in headache [7]; monitor the severity/impact of symptoms in asthma or abnormal vaginal bleeding [8, 9]; or assess short-term responses to treatment [10, 11]. Furthermore, information on short-term symptom trajectories has the potential to provide important prognostic information, and is likely to result in better long-term prediction of health outcomes [12, 13].

Despite a wide variation in musculoskeletal pain trajectories over time, early changes in painful symptoms after prescribing an analgesic (such as an opioid) are not routinely collected as part of musculoskeletal follow-up assessments, which often take place several weeks or even months later [14–16]. Monitoring of these changes in response to opioids is very important, and now underpins current guidelines [17], especially since evidence for the long-term effectiveness of opioids is lacking [18], and there is an increasing recognition of the harms related to opioid use [19, 20]. Although many studies have reported on the long-term (6–12 months) outcomes of musculoskeletal pain [12, 15], and investigated long-term trajectories using repeated pain assessment [15, 21–23], little is known about short-term pain trajectories following primary care consultations, how they relate to long-term outcomes, and in what way they might be used to support the primary care management of musculoskeletal pain.

When pain trajectories are measured, data have often been collected using paper diaries, which are cumbersome, have low completion rates, and may be completed retrospectively resulting in inaccurate and potentially biased data [24–26]. In recent years alternative methods for daily data collection have been proposed, including the use of text messaging [27–29], palm top computers [30], or Smartphone technology [31]. These approaches are gaining popularity given the increasing use of Smartphones and particularly now with two thirds of British adults owning one [32]. One systematic review identified 55 articles reporting the design, evaluation, or use of smartphone-based software for healthcare professionals, students, or patients. The authors highlight the increasing use of Smartphone technology in healthcare and their potential role in patient education, disease self-management, and remote monitoring of patients [33]. Limitations of Smartphone technology have also been reported, most importantly the lack of personalised feedback, usability issues (e.g. ease of data entry), and poor integration of Smartphone data with electronic health records [34, 35]. A systematic review of currently downloadable pain monitoring apps has highlighted the lack of scientific rigour used to ensure validity and reliability with respect to pain measurement [36]. However, where attention to validation has been robust, moderate to high reliability and validity has been reported [37, 38]. Therefore, in this feasibility study, our aim was to:

1. develop a Smartphone Application (“Keele Pain Recorder”) for use by patients with painful musculoskeletal conditions to record daily information on their pain severity and the impact of pain on daily life.
2. assess the acceptability and clinical utility of the Pain Recorder in terms of completion rates, feasibility of its use, and its influence on GP decision-making. Even if an app is found to have a high level of validity in collecting data, its clinical usefulness is only as good as its level of acceptability to the user in day to day use [39]. It is therefore, as part of any app development, essential to examine how acceptable and useful to the user it is.
3. assess face and content validity and explore construct validity of data collection using the Keele Pain Recorder in musculoskeletal patients presenting to primary care receiving new analgesic prescriptions. We hypothesised that single Pain Recorder items were highly correlated with validated questionnaires measuring the same domain of interest i.e. whether there was a strong correlation between the Keele Pain Recorder scores and questionnaire scores at baseline (day 1–3 for the Pain Recorder) and follow-up (last 3 days for the pain app), and between changes over time in scores from the app and questionnaires (longitudinal validity).
4. assess the daily changes in pain and other symptoms (short-term pain trajectories) for patients presenting with a new episode of MSK pain during the first 4 weeks use of the Keele Pain Recorder. This is an area of data collection that is important since information on short-term symptom trajectories might help in establishing the possible cause of symptoms (diagnosis) [7]; estimate the future course of a condition (prognosis) [11, 40]; monitor the severity of symptoms in patients with chronic conditions [9]; and assess early response to treatment (intervention) [10, 11]. However, early changes in pain and other symptoms are often not assessed until several weeks or even months after the first consultation or start of treatment [14–16], and development of the Keele Pain Recorder (KPR) offers a clear opportunity to examine these short-term trajectories in relation to these areas of clinical assessment.

## Methods

### Development of the “pain recorder”

#### Design of the Pain Recorder

A workshop with 9 members of Keele University’s Institute for Primary Care and Health Sciences (IPCHS) Research User Group (RUG) was organised to obtain the views and opinions of people with experience of living with chronic musculoskeletal pain to underpin the design of the app. During the workshop, drafts (mock-ups) of screens which might be potentially used for the Pain Recorder, and mock-up examples of possible pain trajectories were presented to the RUG and a range of aspects of the design and content of the proposed app were discussed. These included: content and phrasing of questions regarding pain and the impact of pain on everyday life to include in the app; response options; appearance and functionality of the app; content of help functions; how completion rates could be optimised. The app developer as well as members of the research team attended the workshop. Consensus on the final content was achieved through the use of electronic voting (using Turning Point) which allowed the RUG to vote independently, ranking options in order of preference [41]. The software calculates a weighted response giving greater importance to higher ranked options and thereby a clear indication of the group’s overall choice on any aspect of the app discussed. Discussions were audio-recorded, and written reports drafted summarising feedback and advice from the RUG. The app developer used the outcomes from this workshop to develop a first alpha-version of the Pain Recorder.

#### Symptom measures

Though no formal reference to IMMPACT pain measurements were made as this was a pain monitoring app that was developed entirely by patients, the content of the app reflects accurately those pain measurements suggested as outcome measures in its recommendations [42]. Consequently, the KPR records data on all domains including pain intensity, analgesic use, the temporal nature of pain, its effect on physical function, emotional functioning (mood), pain trajectories assessing change, and a record of adverse events.

All measures were derived by the RUG and clinical advisory group (CAG). Pain recording was measured using a standard numerical scale (0 = no pain, 10- worse pain imaginable) [43]. Pain impact was recorded in terms of interference of pain with activities at home, leisure, or work. A scale of 1–5 where 0 = none and 5 = extremely was felt most appropriate. This scale was developed by the RUG amended from the SF36 [44]. If the patient had been asleep in the previous 12 h this could be recorded and whether their pain had interfered with sleep or not. This was based on the Jenkins sleep scale [45]. For psychometric assessment the WHO-5 Well-Being Index assessing cheerfulness was used, scored from 0 = none of the time, to 5 = all of the time. The WHO-5 psychometric properties have been assessed and found to have adequate validity in screening for depression and in measuring outcomes in clinical trials. It has good construct validity as a unidimensional scale measuring well-being [46]. The RUG voted against using the ‘pain bothersomeness’ question commonly used in research [47].

A novel suggestion from the RUG related to assessing medication adherence. Rather than recording the number of tablets taken, the group felt this was too complex and time consuming and it was suggested the patient record if they had taken their medication ‘as prescribed’, ‘less than’, or ‘more than’ recommended by their GP. Finally, perceived side effects could be recorded at each data entry point, or at any time the user wished to.

#### Face and content validity, beta-testing

The alpha version of the Pain Recorder was demonstrated, including examples of downloads of pain recordings (trajectories), during further workshops with our patient advisory group (6 RUG members), and an additional clinical advisory group (13 participants) of (academic) GPs, physiotherapists, research nurses, primary care researchers, research facilitators, and an IT manager. The following issues were discussed: (a) content and wording of items included in the Pain Recorder (face validity); (b) whether the app incorporated all relevant aspects of pain interference (content validity); (c) its utility for discussing symptoms and medication use with patients (including adherence and possible adverse effects); (d) its utility for supporting decisions regarding treatment; and (e) opportunities for using the app for research purposes. These meetings were recorded, and written reports produced to summarise comments and suggestions made by the group.

Following these meetings, a beta version of the Pain Recorder app suitable for Android smartphones/tablets was developed by the study team based solely on the recommendations from these groups.

The clinical advisory group (CAG) reviewed the app and overall it was liked. It was felt that it offered the opportunity for GPs to discuss pain control with their patients in a new way that related medication use and activity. An interesting point was made that in fact the app might encourage adherence to decisions made on pain medication use through its repeat recording reminder. It was agreed that all elements were clinically useful and informative. They did not suggest further changes to the context of the app.

Beta testing was undertaken over a four-week period using 6 ‘Pain Recorder’ loaded tablets by study team members, RUG members, and additional members of the public of varying age, educational level, and familiarity with smartphone technology. Following identification of ‘bugs’, typographic errors and any elements of the Pain Recorder that could cause confusion, the developer produced the ‘Gold’ version of the app which was to be used in the feasibility study.

### Feasibility study

#### “Pain recorder” deployment and data collection

##### Study population

Patients aged 18 years and over who consulted at their general practice with a new episode of musculoskeletal pain (defined as no consultation for musculoskeletal conditions in the previous 3 months) were invited to take part in the study, if they were prescribed a stronger class of analgesic (Non-Steroidal Anti-Inflammatory Drug (NSAID) or strong opioid/opioid combination medication containing more than 8 mg of codeine per tablet). Each GP system was programmed to notify the GP when a suitable patient for the study was identified through an appropriate prescription being issued for one of the stronger analgesics or NSAIDs during a consultation. This would also tag the patients record identifying them as a suitable candidate for the study with a searchable code. An automated message would appear on the GP’s computer screen (a ‘pop-up’) during the consultation and accordingly the GP could invite the patient to the study. If the GP did not invite the patient, weekly searches of the GPs computer system identified these individuals through the code and they were then invited to the study via a letter from the GP’s surgery. Patients were excluded by the GP if they had symptoms or signs indicative of pathology requiring urgent medical attention; pain because of cancer or other non-musculoskeletal condition; pain due to an acute injury; inability to read and speak English; vulnerability (e.g. dementia, terminal illness, severe mental health problems); or travelling outside Europe/for longer than 30 days following the consultation. Eligible patients were identified either during the consultation by the GP or nurse practitioner, or after the consultation through regular searches of consultation records. If the patient was considered suitable, the patient was informed about the study and provided with a participant information sheet and referral form. Patients signing this form provided written consent to be contacted by the research nurse. The GP/nurse practitioner prescribed pain medication as planned and continued to provide care as usual during the study. Four general practices in North Staffordshire participated in the study. Ethical approval for this study was obtained from the NRES Committee West Midlands (REC Reference: 14/WM/1214). Patients involved in the RUG group and workshops after data collection received travelling expenses whilst the GPs involved and users of the app in the study were not remunerated.

##### Data collection

The research nurse contacted patients interested in the research project, provided further information about the study and made an appointment for a baseline visit at the general practice. When signed informed consent was obtained, participants completed a baseline questionnaire, which included questions on sociodemographic variables, lifestyle factors (alcohol use, physical activity level); height and weight (for Body Mass Index, BMI); history of musculoskeletal pain; use of analgesics; and baseline levels of pain intensity, pain interference, sleep, and mood using validated questionnaire items. The baseline pain assessment recorded how long the patient had been experiencing pain (less than 2 weeks to more than 12 month), and how long since they had had no pain for more than a month. The main measure relating to the app was pain severity in the last 24 h.

The research gave the participant’s a tablet pre-loaded with the app for the duration of the study (4 weeks). The Pain Recorder was demonstrated and set up for the participant, who was then invited to enter recordings twice daily for a period of 4 weeks.

An appointment was made for the participant to attend a follow-up meeting with the research nurse and a repeat consultation with the GP or nurse prescriber 1 month after receiving the Pain Recorder. During the follow-up meeting the research nurse exported anonymised data from the Pain Recorder as a password protected file and sent this to a secure NHS account, accessible only by the GP, the research nurse and members of the study team responsible for data management and analysis. Graphical presentations of the data were produced allowing the GP or nurse prescriber to discuss the pain trajectories and response to prescribed pain medication with the participant. The GPs and Nurse practitioners received no formal training in how to interpret the graphs, or use them in their management. This allowed us to examine how this novel data collection method would be used, or not used, in their day to day practice. The participant was given a follow-up questionnaire to be completed at home, including follow-up questions on pain intensity, pain impact, sleep and mood; course of pain over the past month, acceptability of the Pain Recorder, and the participants’ views regarding the use of the Pain Recorder in the management of their pain problem.

#### Acceptability, usability and clinical utility

Acceptability was assessed by descriptively summarising Pain Recorder completion rates and responses by study participants to questions regarding acceptability and usefulness in the follow-up questionnaire. Furthermore, after completion of data collection a workshop was organised with study participants, which was facilitated by members of our RUG, and semi-structured telephone interviews were conducted with GPs from recruiting practices to discuss: (a) acceptability and feasibility of using the Pain Recorder in clinical practice; (b) its utility for discussing symptoms and medication use; (c) its utility for supporting treatment decisions; (d) opportunities for using the Pain Recorder for research purposes and (e) its usability in terms of ease of use and interference with patients’ daily activities. Usability is a key strength of any smartphone application as it will ultimately determine whether the app will be used in daily practice by patients. The full potential of any app is unlikely to be realized unless the development and design take into account usability [39]. The workshop group with users of the app and RUG members followed an open forum. The meetings were led by the RUG members who had developed the content of the app using headline topics that were then detailed using the thinking aloud technique. The agenda here was driven by patients, and reflects their experiences with no input from clinicians such that the perspectives that evolved were personal to users of the app. GPs interviews followed a specific set of questions relating to their interaction with the user and were purely their own opinion.

Workshop discussions and interviews were audio-recorded, and reports written to summarise responses to questions and feedback provided during discussions.

#### Initial testing of construct validity

The pain trajectories generated by the app were downloaded and assessed by a second clinical advisory group (CAG) to discuss the validity of recordings and determine if subgroups of participants with distinct patterns of short term pain could be identified.

Construct validity was explored by comparing scores for pain, sleep interruption, and mood collected at baseline and one-month follow-up using validated questionnaires with entries on the Pain Recorder. The questionnaires used the same validated questions as those used in the app, for example the WHO 5 well-being index [46], a RUG adapted SF36 pain interference scale and numerical pain rating scale [43]. Other validated questions included in the questionnaires included pain duration [48], pain trajectories [47], the Jenkin’s sleep questionnaires [45], and physical activity (GPAQ) [49].

##### Statistical analysis

Descriptive statistics were used to characterise the study population, in terms of age, gender, BMI, work status, pain characteristics, global health and lifestyle factors. Potential subgroups of participants with distinct patterns of short-term pain were identified using blind voting. Nine members, including GPs, a pain consultant, and physiotherapists were shown trajectories of pain intensity, pain interference and mood for each participant, with blind voting (Turning Point software) being used to identify distinct trajectories and classify participants according to these proposed subsets. Spearman’s correlation coefficient was used to assess the strength of correlations between Pain Recorder scores and questionnaire scores.

#### The ‘gold’ version of the Keele pain recorder app

The final version of the app had an alarm built in to remind the user to record their pain experience at 8 am and 8 pm. This could not be switched off, but the tablet/phone could be if the patient did not wish to be disturbed. There was an initial set up which was completed with the research nurse to demonstrate the app. The patients personal ID was entered and in the set-up gender and date of birth were recorded. Then each screen subsequently appeared to ask in order about (1) Average pain level in the last 24 h (2) Level of pain interference or whether their pain disturbed sleep (3) Well-being questions. At the set alarm times the patient would then complete a similar set of questions (1) Average pain in the last 12 h (2) Level of pain interference or had the pain disturbed sleep (3) Well-being questions (4) Medication use (more than, less than or as prescribed) (5) Did the patient feel they has side effects and if yes a diary record of these could be entered (date and time recorded) (6) A screen asking if they wanted to record anything else and if ‘yes’ they could enter a written note (date and time recorded). The patient had the option to enter a pain recording at any other time they wished other than the pre-set times.

From the front page of the app patients could access a help section for each page, giving advice on completing the page, and this could be accessed from each page when being competed as well. There was also in this section a Frequently Asked Questions (FAQ) page which answered questions such as who to contact if there is a problem, and who had access to the information recorded (Screenshot 8, Additional file 1). The last page of the app advised patients if they felt severely unwell during the study for any reason to contact urgent medical help, ensure the tablet/phone was charged at least once per day, if the patients missed a recording not to worry and just complete the next.

After 1 month the patient returned to the Research Nurse on the day of their appointment with their GP, the graphical output from the app was downloaded and passed to the GP for use in the consultation. The follow up questionnaire was completed.

## Results

### Development and testing of the “pain recorder”

The ‘gold’ version of the Pain Recorder developed following the RUG and CAG workshops consisted of baseline information; 6 questions to assess pain severity, impact of pain on sleep or activities during the day, mood, use of analgesics, and experience of adverse effects from analgesics; two help functions and a diary. Screenshots are available in Additional file 1 (Screenshot 1–7). The RUG felt that it would not be onerous for users to record data twice per day, every day (between 8 and 10, am and pm), and that an alarm would be helpful to remind them.

The RUG felt that baseline information should be kept to a minimum to improve completion rates and consisted of age, gender and number of days per week where the user was physically active for more than 30 min per day.

The RUG indicated that it was imperative instructions for all sections were easily accessible and written in plain English. It was also felt that a frequently asked questions (FAQ) section would be helpful, and in this section information about contacting the study team should be easily accessible.

One suggestion from the RUG was to link physical activity, events, and other unusual activities to the recordings as this might explain strong fluctuations in pain trajectories. The conclusion was to allow patients access to a diary which would record the date of entry and in which such information, and any other that the patient felt necessary, could be recorded and made available to the GP.

Beta testing of the app revealed 38 ‘bugs’ and issues that required remedy by the developer. The majority of these were phraseology within the instructions and help sections in the app, two major ‘bugs’ concerned functionality (e.g. pressing ‘help’ on the side effects page led to the diary and not the appropriate help page). Correction of these errors produced the final ‘Gold’ version which was used in the feasibility study.

Finally, the study team and developer produced instructions for installing the Pain Recorder onto Smartphones, how to complete the app, and how to download pain recordings for both the research nurses and patients to use. Downloads from the app could be copied and pasted into GP medical records, printed for the GP to use with the patient in a follow up consultation, as well as emailed to the GP through a secure NHS.net account used by the research nurse.

### Acceptability and clinical utility of the “pain recorder”

#### Completion rates

Five general practices (17 general practitioners, 2 nurse practitioners) agreed to take part in the Keele Pain Recorder study, four of which recruited participants to the study. The computer-generated message identifying potential candidates for the study (pop-up) fired on 167 occasions from which 27 suitable patients were invited to the study and 25 consented to take part. Three withdrew consent and one was lost to follow up when they did not attend the baseline clinic. Of the 21 participants, 13 were females and 8 males, with a median age of 62 (IQR 50 to 70) years old (Table 1). Their median baseline pain intensity was 6 (IQR: 4–7). Eighteen participants attended the follow up clinic and returned the 1-month follow-up questionnaire with three being lost to follow up at this point. The participants entered 862 records, 53.1% in the morning, and 46.9% in the evening. Of these records, 255 (30%) were entered during the first week, 198 (23%) the second week, 198 (23%) the third week, and 211 (24%) the fourth week. Median number of records per participant were 23 in the morning and 22 in the evening over the recording period of 4 weeks, indicating that recordings were made on 73.3% of days. There was no association between completion rates and gender or baseline pain intensity levels, but older participants tended to record more often than younger participants (Spearman correlation coefficient 0.47, *p* = 0.03).

**Table 1:** Patients self-reported characteristics at baseline

	Total participant (*n* = 21)
Demographic characteristics	
Female, n (%)	13 (61.9)
Age, median (IQR)	62 (50, 70)
Body mass index, median (IQR)	27.8 (23.1, 32.0)
Alcohol drink days per week, median (IQR)	1 (0, 2)
Work status, n (%)	
Working full-time in a paid job	7 (33.3)
Working part-time in a paid job	3 (14.3)
Employed but currently off sick	0 (0.0)
Housewife/husband	1 (4.8)
Unemployed due to pain condition	0 (0.0)
Unemployed for other health reasons	1 (4.8)
Retired	7 (33.3)
Student	0 (0.0)
Other	1 (4.8)
Missing	1 (4.8)
Pain characteristics	
Intensity last 24 h (0–10 scale, median (IQR))	6 (4, 7)
Location of current pain ^a^, n (%)	
Neck	2 (9.5)
Low back	8 (38.1)
Shoulder	3 (14.3)
Hip	7 (33.3)
Elbow	2 (9.5)
Knee	3 (14.3)
Wrist or hand	6 (28.6)
Ankle or foot	2 (9.5)
Other	1 (4.8)
Pain duration of current episode	
< 2 weeks	6 (28.6)
2–6 weeks	6 (28.6)
7–12 weeks	2 (9.5)
3–6 months	3 (14.3)
7–12 months	2 (9.5)
> 12 months	2 (9.5)
General health, n (%)	
Excellent	2 (9.5)
Very good	7 (33.3)
Good	10 (47.6)
Fair	2 (9.5)
Poor	0 (0.0)

*IQR* interquartile range

^a^Subgroups are not mutually exclusive as more than one location could be selected

Table 2 gives a summary of results from the 1-month follow-up questionnaire (response *n* = 18; 86%). All participants found the Pain Recorder easy to read, with the majority using the app daily or often. Six participants reported that the use of the Pain Recorder had interfered with their daily routine. The majority discussed the graphs with their GP, and 11 reported that the GP showed interest in the results. Most found the graphs easy to understand, but opinions varied regarding their impact on helping to discuss their pain or changes on medication.

**Table 2:** Summary of results from the follow-up questionnaire

	n	n	n
Frequency of app use	daily: 9	often: 9	sometimes/never: 0
Interference with daily routines	No: 12	Yes: 6	
Easy to read	Yes: 18	No: 0	
Use of HELP function	No: 16	Yes: 2	
Contact with research nurse	No: 14	Yes: 4 (issues resolved)	
Graphs discussed with GP	Yes: 14	No: 3	Missing: 1
GP showed interest	Yes: 11	No: 4	Missing: 2
Graphs difficult to understand	Not at all: 8	Little bit: 3	Very: 1
Graphs helpful to discuss pain and impact	Very: 4	Somewhat: 5	Not at all: 3
Impact of graphs on pain medication	Stopped/changed: 2	Continued: 6	No influence: 6

No technical errors within the app occurred during testing other than with two users where we were unable to download the graphical output at the follow up nurse research clinic, and these were later downloaded and given directly to the GP who consulted the patient in the following week.

#### Workshop with participants

Two users attended the workshop with 2 RUG members who developed the app. All study participants who attended the participants’ meeting indicated that the Pain Recorder had been very easy to use (‘child’s play’), self-reporting that it took only 2 min on average to complete (max 5 min), and not interfering with daily life or sleep. They had not needed the ‘Help Function’, nor had required help from the study team or other people (these two users were not those who reported it did interfere or who had used the help function in the questionnaires). There was a discussion regarding the usefulness of the diary function: although this was considered helpful to provide context to pain impacting on specific activities, it was not needed on a daily basis. In terms of utility the Pain Recorder was perceived as a tool to inform the GP how they had been managing their pain, and it contributed to decisions by the GP regarding medication changes. They felt the graphs were useful in aiding the discussion and understanding of their pain. They also indicated that the app did not directly influence their thoughts, feelings or actions related to mood, pain interference, or medication usage. Important suggestions for future use of the Pain Recorder included (1) to make data recorded by users available to them in graphical output at any time, and (2) to make the app a ‘real time’ monitoring tool which might offer advice on treatment when required, either from protocols in the app or from a medical professional.

#### Results from GP interviews

Results from semi-structured telephone interviews (20–30 min each) with one GP from each of the four recruiting practices showed that they felt the graphs generated by the Pain Recorder were easy to interpret, and most felt that the graphs were useful in helping patients make choices about their use of medication. GPs were confident that patients were happy to bring these graphs to the consultation in the expectation of discussing the results with their GP. Two GPs however felt they could get the same information from taking a history from the patient, and only one felt it influenced their management strategy of the patients’ conditions. One GP who did not use the graphs in the consultation did not do so as they felt there was not enough time to do this, so chose to ignore them. All four GPs said they would recommend the Pain Recorder to patients for self-monitoring of their condition. In terms of usefulness for research, two felt it would be more useful in investigating and managing chronic rather than acute pain, whilst one GP thought it had a place in trials of analgesics to monitor patients’ response. They suggested that in future iterations patients should be able to see trends in their pain levels at any time they wished.

### Face and construct validity of the “pain recorder”

At both baseline and follow-up (4 weeks), the correlations of pain intensity scoring between questionnaire and Pain Recorder were very strong (Spearman’s correlation coefficient ≥ 0.79, *P* <  0.0001). For pain interference and mood, although weaker, significant or potential correlations were still seen based on this relatively small group of participants (Table 3). The correlation was even weaker for sleep where the question examined related to ‘trouble staying asleep’. The number of entries in this category were fewer with missing data and probably accounts from some of the issues. Overall there were 862 responses in total. 840/862 (97.5%) responded with prescribing information. Among the 862, in total 93(10.7%) reported a side effect. Those using analgesia “as prescribed” (*n* = 585) and “less than prescribed” (*n* = 186) had similar side effect rates (10.0 and 11.9%, respectively). Side effect rate appeared to be higher in “more than prescribed”, but there were only 6 such records. No significant difference of side effect was observed between the three (*P* <  0.33).

**Table 3:** Correlation between Pain Recorder and questionnaire scores for assessing construct validity

Pair of variable	Spearman’s correlation coefficient (r)	*P* value
1. Baseline questionnaire pain intensity & Pain Recorder pain intensity first 1–3 ^a^ days (*n* = 20)	0.79	< 0.0001
2. Baseline questionnaire pain interference & Pain Recorder pain interference first 1–3 ^a^ days (*n* = 20)	0.60	0.005
3. Baseline questionnaire mood & Pain Recorder mood first 1–3 ^a^ days (*n* = 20)	0.27	0.27
4. Baseline questionnaire “staying asleep” & Pain Recorder “staying asleep” first 1–3 ^a^ days (*n* = 14)	0.22	0.45
5. Follow-up questionnaire pain intensity & Pain Recorder pain intensity last 1–3 ^a^ days (*n* = 18)	0.92	< 0.0001
6. Follow-up questionnaire pain interference & Pain Recorder pain interference last 1–3 ^a^ days (*n* = 18)	0.40	0.11
7. Follow-up questionnaire mood & Pain Recorder mood last 1–3 ^a^ days (*n* = 18)	0.15	0.56
8. Follow-up questionnaire “staying asleep” & Pain Recorder “staying asleep” last 1–3 ^a^ days (*n* = 15)	0.53	0.04

^a^Averaged score or category

### Assessment of pain trajectories from the “pain recorder”

**Fig. 1:**
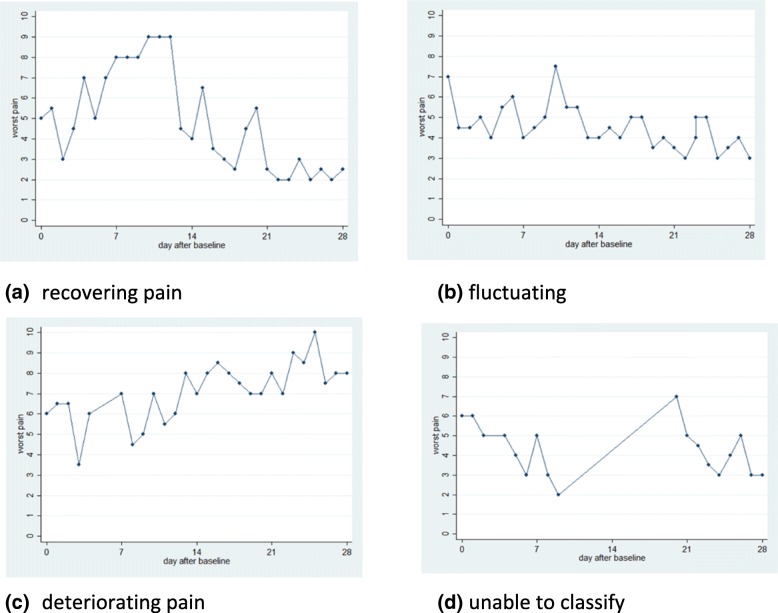
Real life examples of different pain 4-week trajectories pain recorded by the app: (**a**): recovering; (**b**) fluctuating low to moderate pain; (**c**) deteriorating; (**d**) unable to classify

A wide variety of short-term pain trajectories were evident from recordings obtained from participants of the feasibility study. The clinical advisory group proposed to classify these into four main groups (Fig. 1): (a) recovering (five participants); (b) fluctuating low to moderate level of pain (eight participants); (c) deteriorating; (d) unable to classify (two participants), when there was more than 7 days of consecutive data missing.

**Fig. 2:**
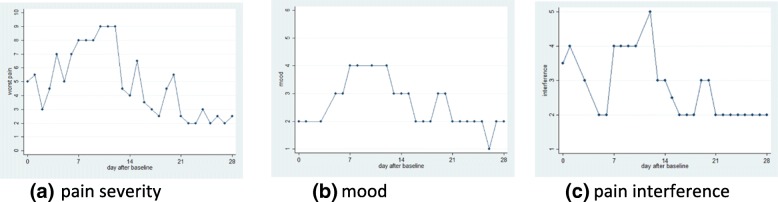
Real life changes in pain score recorded by the app (**a**) (1 = no pain, 10 = worst pain), mood (**b**) (1 = cheerful all the time 5 = none of the time) and interference (**c**) (1 = not at all, 5 = all the time) over a 28 day period following initiation of a new prescription analgesic

This CAG visually inspected the temporal associations between changes in daily pain score, mood, and pain interference (for example Fig. 2). The subjective impression in this example was that the level of pain was reflected in scores for mood score and interference of pain on everyday activities, suggesting face validity of the pain trajectories.

## Discussion

In close collaboration with patient representatives and clinical stakeholders, we developed a smartphone/tablet app, which can be used to help clinicians and patients monitor painful musculoskeletal conditions in response to analgesic prescribing. Early testing in a small sample of people consulting with musculoskeletal pain in general practice showed promising results in terms of face and content validity, acceptability, and clinical usefulness.

Despite there being more than 270 pain apps available to download [50], there have been few studies that have validated pain monitoring apps. Often existing apps are designed by software engineers and there is little input from patients and clinicians in the design and evaluation of these apps [50, 51]. A recent review and Editorial examining pain app design and use highlighted this fact, particularly the finding that apps sometimes appear to offer solutions to pain management with little thought given to the content of the app being validly related to clinical factors [31, 52]. This same review concluded that the development of apps needs to be evidence-based with rigorous evaluation of outcomes being important in enhancing the understanding of the potential of these apps [52]. Our study directly addresses this issue in that the design of the Keele Pain Recorder was driven by both patient and clinician experience and advice, whilst its clinical usefulness, acceptability and validity was assessed by several primary care clinicians in conjunction with the app users. One recent study has offered a more vigorous examination of a pain monitoring App. Suso-Ribera found that weekly monitoring of pain over a 30-day period found similarly high levels of compliance, acceptability, ease of use and construct validity to the KPR [53]. Jamison also found that a pain monitoring app was acceptable to patients and easily utilised [54]. This is encouraging as it suggests that pain monitoring apps in varying forms are devices that patients will be willing to engage with when managing their pain.

A series of iterative workshops and interviews with patients, clinicians and musculoskeletal researchers were used to (i) discuss content and functionality of the app, (ii) further improve the design of the app, and (iii) explore opinions regarding acceptability, validity, and usefulness. Patients confirmed that they felt discussing recordings from the app helped with their GP’s understanding of their pain condition, whilst GPs considered it useful in helping their patients make choices about medication. Patients found the Keele Pain Recorder easy to use, and the GP found the graphical output easy to interpret. As in a study from the USA, both groups found using a pain recorder app acceptable in clinical practice [51].

The individual pain trajectories of patients varied widely, reflecting wide differences in the impact and experience of pain, even over the course of only 1 month after consulting in primary care. However, the trajectories were determined through a visual analysis and a consensus exercise amongst experienced clinicians rather than using statistical methods. The number of trajectories available to examine were too few and precluded this. Therefore, it is possible that the trajectories determined were subject to individual bias, however, agreement in each case was by majority, and each participant had extensive experience in the management of musculoskeletal pain. There may be value in assessing early symptom trajectories in people with musculoskeletal conditions, though further research is needed before any potential clinical usefulness can be established. Many prognostic models developed in studies of low back pain have been shown to have limited predictive performance [55], although some tools, such as the Start Back Screening Tool, have been extensively tested and are now also available as a smartphone app. Although its predictive performance has been confirmed in several populations, one study showed that it was no better than clinical acumen in predicting low back pain outcomes [21]. One reason for limited predictive performance of prognostic tools may be that most are based on only a single assessment of pain. In our study, the Keele Pain Recorder app demonstrated three main short-term pain patterns: improving, fluctuating or worsening, which reflect those previously reported in patients with low back pain [6, 22, 23]. Potentially the use of a smartphone/tablet pain app might allow for more frequent and detailed characterisation of pain trajectories shortly after healthcare consultation. This, therefore, might be used in the future to help develop more accurate predictive models and early identification of patients likely to do well (preventing unnecessary treatment) versus those who may benefit from early, more intensive treatment [15].

An important limitation is the small sample size of the feasibility study, which limits generalisability and precision of our findings regarding construct validity. Though the sample was small, we found a good correlation between established and validated pain measures in the baseline and follow-up questionnaires. However, there was no significant correlation with interference, mood and sleep. It is possible that in all these 3 domains (mood, interface and sleep), this might have occurred because we averaged results from the app over the first and last 3 days of the study. Therefore, due to the potential variability in these factors recorded in the app over this time, they may not reflect those recordings in the baseline and follow-up questionnaire. Small numbers in the study will also have limited our power to detect any correlation. Further testing of the Pain Recorder in larger groups of patients is needed to more formally and quantitatively investigate construct validity of the app, and to establish its clinical utility for monitoring pain by investigating impact of its use on clinical decision making and patient outcomes. Additionally, we only tested the app amongst patients with musculoskeletal pain, so the generalisability of its use in other conditions such as headache or pelvic pain cannot be assured. Similarly, our study focused on monitoring pain following prescription of a stronger class of analgesics, but the app could also be used to study pain trajectories following other types of treatments for pain.

Assessing acceptability and usability of the KPR presents problems in common with other smartphone apps [56]. Both factors are interdependent, and it is likely that testing these elements 1 week after starting using the app would provide different results to our assessment which was at 1 month of use. There may be issues for users when they first use the software due them being unfamiliar with it, and therefore limit ease of use. Consequently, acceptance of the app might be diminished. However, after a month’s use, familiarity with the mechanics of the software might improve usability for the patients. Equally though, with loss of novelty, the user might lose interest in it and so its acceptance as a daily activity might be lost. Future research might overcome these issues through testing at both points in time to give a more comprehensive view of an apps acceptability and usability.

Low completion rates were a limiting factor in this study. This might have been compounded by the intrusion of the technology into daily life with it being perceived as an interference in the user’s normal routine. There are ways in which this might be overcome, for example gamification has been shown to improve engagement and retention in app use [57]. Equally if the app had been used on the patient’s own mobile smartphone, the more immediate access to this device (rather than a tablet kept elsewhere) might have improved completion rates. An additional limitation relates to the comparison of the baseline and follow-up questionnaire pain scores with those recorded in the app to determine how valid these were. The most valid figure would have been to equate the single baseline/follow-up figure with the first and last day score in the app giving a direct contemporaneous comparison. However, we chose to use the initial and final 3-day average of the study. This might have led to errors in the comparison due to the potential variability of the patient’s pain during that period when compared to the single recording at baseline and follow-up. However, due the possibility that we might recruit low total numbers to this novel research, we chose to use the 3-day average which would potentially give at least 1 record during the 3 days. If we had only used the 1st or last day alone, there might have been more missing values.

Two recordings could not be classified due to a lack of consecutive data (> 7 days). This limitation was not overcome by using reminder on the tablet and is likely to be due to external circumstances, or interference of the technology in the participants’ everyday life, which was indicated to occur sometimes according to 6 of 18 participants in the feasibility study. This may have been compounded by the fact that the users did not have direct access to their pain graphs, which might have acted in a positive way to reinforce use of the app. Concerns have been expressed regarding the potential negative impact of frequent pain reporting on physical health and work productivity [58]. When asked specifically, participants reported that they felt using the app had not directly influenced their thoughts, feelings or actions related to mood, pain interference, or medication usage. However, further research should investigate to what extent the use of the Keele Pain Recorder is associated with consultation rates, healthcare resource use, and changes in physical or mental health.

We developed secure methods for archiving, downloading and emailing pain trajectories from the Pain Recorder to the GP and patient to be used in their consultation. These methods will now be extended to allow open-access to the Pain Recorder and support use of the app on both Android and Apple phones or tablets [59, 60]. Future research, however, needs to examine how such data may be accessed in a ‘live’ format such that GPs or other health care professionals may use information regarding pain trajectories to manage a patient’s condition when it deteriorates, for example during an acute attack of gout or a flare of knee osteoarthritis. Research may also focus on the potential of using the Keele Pain Recorder in self-management, such that software might independently recognise when a patient is at risk of developing disabling pain, offering feedback and advice to the patient without the input of a third party such as the GP. However, these devices will require rigorous assessment to ensure the advice is safe, relevant, and does not miss the possibility of ‘red-flag’ conditions such as cancer pain or other conditions (e.g. inflammation) that need medical attention.

## Conclusions

In conclusion, within this limited sample of users, we have successfully developed the Keele Pain Recorder tablet app which both patients and clinicians considered easy to use. Early testing shows promise in terms of validity, acceptability and clinical usefulness, with clear priorities identified for further testing and investigation of its potential role and impact in the clinical and self-management of musculoskeletal conditions and other pain problems.

## Additional file

Additional file 1: Keele Pain Recorder Screenshots. Screenshots of data capture points, help page, and frequently asked question (FAQ) page in the Keele Pain Recorder App. (DOCX 408 kb)

## Abbreviations

BMI: Body mass index
CAG: Clinical advisory group
FAQ: Frequently asked questions
GP: General practitioner
IPCHS: Institute for primary care and health sciences
KPR: Keele pain recorder
NIHR: National Institute for Health Research
NSAID: Non-steroidal anti-inflammatory drug
RUG: Research User Group

## References

[1] Jordan KP, Jöud A, Bergknut C, Croft P, Edwards JJ, Peat G, Petersson IF, Turkiewicz A, Wilkie R, Englund M (2014). International comparisons of the consultation prevalence of musculoskeletal conditions using population-based healthcare data from England and Sweden. Ann Rheum Dis.

[2] Ndlovu M, Bedson J, Jones PW, Jordan KP (2014). Pain medication management of musculoskeletal conditions at first presentation in primary care: analysis of routinely collected medical record data. BMC Musculoskelet Disord.

[3] Muller S, Bedson J, Mallen CD (2012). The association between pain intensity and the prescription of analgesics and non-steroidal anti-inflammatory drugs. Eur J Pain.

[4] Perrot S, Concas V, Allaert F, Laroche F (2008). Deciding on analgesic prescription dosing for acute back pain: once daily or more?. Presse Med.

[5] Hawker GA, Mian S, Kendzerska T, French M (2011). Measures of adult pain: Visual Analog Scale for Pain (VAS Pain), Numeric Rating Scale for Pain (NRS Pain), McGill Pain Questionnaire (MPQ), Short-Form McGill Pain Questionnaire (SF-MPQ), Chronic Pain Grade Scale (CPGS), Short Form-36 Bodily Pain Scalee (SF-36 BPS), and measure of Intermittent and Constant Osteoarthritis Pain (ICOAP). Arthritis Care Res.

[6] Dunn KM, Campbell P, Jordan KP (2013). Long-term trajectories of back pain: cohort study with 7-year follow-up. BMJ Open.

[7] Headache Classification Subcommittee of the International Headache Society (2004). The International Classification of Headache Disorders: 2nd edition. Cephalagia.

[8] Mikolajczyk RT, Buck Louis GM, Cooney MA, Lynch CD, Sundaram R (2010). Characteristics of prospectively measured vaginal bleeding among women trying to conceive. Paediatr Perinat Epidemiol.

[9] National Institute for Health and Clinical Excellence: Asthma. Quality statement 3: Monitoring asthma control. https://www.nice.org.uk/guidance/qs25/chapter/Quality-statement-3-Monitoring-asthma-control. Accessed 2nd Oct 2018.

[10] Leboeuf-Yde C, Rosenbaum A, Axén I, Lövgren PW, Jørgensen K, Halasz L, Eklund A, Wedderkopp N (2009). The Nordic subpopulation research Programme: prediction of treatment outcome in patients with low back pain treated by chiropractors - does the psychological profile matter?. Chiropr Osteopathy.

[11] Chesterton LS, Lewis AM, Sim J, Mallen CD, Mason EE, Hay EM, van der Windt DA (2013). Transcutaneous electrical nerve stimulation as adjunct to primary care management for tennis elbow: pragmatic randomised controlled trial (TATE trial). BMJ.

[12] Dunn KM, Croft PR (2006). Repeat assessment improves the prediction of prognosis in patients with low back pain in primary care. Pain.

[13] Mansell G, Jordan KP, Peat GM, Dunn KM, Lasserson D, Kuijpers T, Swinkels-Meewisse I, van der Windt DA (2017). Brief pain re-assessment provided more accurate prognosis than baseline information for low-back or shoulder pain. BMC Musculoskelet Disord.

[14] Bot SD, van der Waal JM, Terwee CB, van der Windt DA, Scholten RJ, Bouter LM, Dekker J (2005). Predictors of outcome in neck and shoulder symptoms: a cohort study in general practice. Spine (Phila Pa 1976).

[15] Dunn KM, Jordan K, Croft PR (2006). Characterizing the course of low Back pain: a latent class analysis. Am J Epidemiol.

[16] Verkleij SPJ, Hoekstra T, Rozendaal RM, Waarsing JH, Koes BW, Luijsterburg PAJ, Bierma-Zeinstra SMA (2012). Defining discriminative pain trajectories in hip osteoarthritis over a 2-year time period. Ann Rheum Dis.

[17] Faculty of Pain Medicine: Opioids Aware: A resource for patients and healthcare professionals to support prescribing of opioid medicines for pain. 2017. https://www.rcoa.ac.uk/faculty-of-pain-medicine/opioids-aware. Accessed 19th Oct 2017.

[18] Chou R, Turner JA, Devine EB, Hansen RN, Sullivan SD, Blazina I, Dana T, Bougatsos C, Deyo RA (2015). The effectiveness and risks of long-term opioid therapy for chronic pain: a systematic review for a national institutes of health pathways to prevention workshop. Ann Intern Med.

[19] Saunders KW, Dunn KM, Merrill JO, Sullivan M, Weisner C, Braden JB, Psaty BM, Von Korff M (2010). Relationship of opioid use and dosage levels to fractures in older chronic pain patients. J Gen Intern Med.

[20] Dunn KM, Saunders KW, Rutter CM, Banta-Green CJ, Merrill JO, Sullivan MD, Weisner CM, Silverberg MJ, Campbell CI, Psaty BM, Von Korff M (2010). Opioid prescriptions for chronic pain and overdose. Ann Intern Med.

[21] Kongsted A, Andersen CH, Hansen MM, Hestbaek L (2016). Prediction of outcome in patients with low back pain – a prospective cohort study comparing clinicians’ predictions with those of the start Back tool. Man Ther.

[22] Tamcan O, Mannion AF, Eisenring C, Horisberger B, Elfering A, Müller U (2010). The course of chronic and recurrent low back pain in the general population. Pain.

[23] Axén I, Leboeuf-Yde C (2013). Trajectories of low back pain. Best Pract Res Clin Rheumatol.

[24] Lenderking WR, Hu M, Tennen H, Cappelleri JC, Petrie CD, Rush AJ (2008). Daily process methodology for measuring earlier antidepressant response. Contemp Clin Trials.

[25] Okupa AY, Sorkness CA, Mauger DT, Jackson DJ, Lemanske RF (2012). Daily diaries vs retrospective questionnaires to assess asthma control and therapeutic responses in asthma clinical trials: is participant burden worth the effort?. Chest.

[26] Perry L, Kendrick D, Morris R, Dinan S, Masud T, Skelton D, Iliffe S, for the ProAct65+ Study Team (2012). Completion and return of fall diaries varies with participants’ level of education, first language, and baseline fall risk. J Gerontol Ser A Biol Med Sci.

[27] Macedo LG, Maher CG, Latimer J, McAuley JH (2012). Feasibility of using short message service to collect pain outcomes in a low back pain clinical trial. Spine (Phila Pa 1976).

[28] Johansen B, Wedderkopp N (2010). Comparison between data obtained through real-time data capture by SMS and a retrospective telephone interview. Chiropr Osteopathy.

[29] Kent P, Kongsted A (2012). Identifying clinical course patterns in SMS data using cluster analysis. Chiropr Man Ther.

[30] Allena M, Cuzzoni MG, Tassorelli C, Nappi G, Antonaci F (2012). An electronic diary on a palm device for headache monitoring: a preliminary experience. J Headache Pain.

[31] Alexander JC, Joshi GP (2016). Smartphone applications for chronic pain management: a critical appraisal. J Pain Res.

[32] Ofcom: The UK is now a smartphone society. 2015. https://www.ofcom.org.uk/about-ofcom/latest/media/media-releases/2015/cmr-uk-2015. Accessed 19th Oct 2017.

[33] Mosa ASM, Yoo I, Sheets L (2012). A systematic review of healthcare applications for smartphones. BMC Med Inform Decis Mak.

[34] Ramanathan N, Swendeman D, Comulada WS, Estrin D, Rotheram-Borus M (2012). Identifying preferences for mobile health applications for self-monitoring and self-management: focus group findings from HIV-positive persons and young mothers. Int J Med Inf.

[35] El-Gayar O, Timsina P, Nawar N, Eid W (2013). Mobile applications for diabetes self-management: status and potential. J Diabetes Sci Technol.

[36] de la Vega R, Miró J (2014). mHealth: a strategic field without a solid scientific soul. A systematic review of pain-related apps. PLoS One.

[37] Stinson JN, Jibb LA, Nguyen C, Nathan PC, Maloney AM, Dupuis LL, Gerstle JT, Hopyan S, Alman BA, Strahlendorf C, Portwine C, Johnston DL (2015). Construct validity and reliability of a real-time multidimensional smartphone app to assess pain in children and adolescents with cancer. Pain.

[38] de la Vega R, Roset R, Castarlenas E, Sánchez-Rodríguez E, Solé E, Miró J (2014). Development and testing of Painometer: a smartphone app to assess pain intensity. J Pain.

[39] Reynoldson C, Stones C, Allsop M, Gardner P, Bennett MI, Closs SJ, Jones R, Knapp P (2014). Assessing the quality and usability of smartphone apps for pain self-management. Pain Med.

[40] Axén I, Rosenbaum A, Robech R, Larsen K, Leboeuf-Yde C (2005). The Nordic Back pain subpopulation program: can patient reactions to the first chiropractic treatment predict early favorable treatment outcome in nonpersistent low Back pain?. J Manip Physiol Ther.

[41] Turning technologies software: Assess, Monitor and Measure Progress. 2017. https://www.turningtechnologies.eu/turningpoint/. Accessed 23 Oct 2017.

[42] Dworkin RH, Turk DC, Farrar JT, Haythornthwaite JA, Jensen MP, Katz NP, Kerns RD, Stucki G, Allen RR, Bellamy N, Carr DB, Chandler J, Cowan P, Dionne R, Galer BS, Hertz S, Jadad AR, Kramer LD, Manning DC, Martin S, McCormick CG, McDermott MP, McGrath P, Quessy S, Rappaport BA, Robbins W, Robinson JP, Rothman M, Royal MA, Simon L, Stauffer JW, Stein W, Tollett J, Wernicke J, Witter J, IMMPACT (2005). Core outcome measures for chronic pain clinical trials: IMMPACT recommendations. Pain.

[43] Breivik H, Borchgrevink PC, Allen SM, Rosseland LA, Romundstad L, Breivik Hals EK, Kvarstein G, Stubhaug A (2008). Assessment of pain. Br J Anaesth.

[44] RAND Health: 36-Item Short Form Survey (SF-36). https://www.rand.org/health/surveys_tools/mos/36-item-short-form.html. Accessed 21st Sept 2018.

[45] Jenkins CD, Stanton B, Niemcryk SJ, Rose RM (1988). A scale for the estimation of sleep problems in clinical research. J Clin Epidemiol.

[46] The World Health Organisation: The World Health Organisation- Five Well-Being Index (WHO-5). https://www.corc.uk.net/outcome-experience-measures/the-world-health-organisation-five-well-being-index-who-5/. Accessed 21st Sept 2018.

[47] Dunn KM, Croft PR (2005). Classification of low back pain in primary care: using “bothersomeness” to identify the most severe cases. Spine (Phila Pa 1976).

[48] Dunn KM, Croft PR (2006). The importance of symptom duration in determining prognosis. Pain.

[49] Wanner M, Hartmann C, Pestoni G, Martin BW, Siegrist M, Martin-Diener E (2017). Validation of the Global Physical Activity Questionnaire for self-administration in a European context. BMJ Open Sport Exerc Med.

[50] Lalloo C, Jibb LA, Rivera J, Agarwal A, Stinson JN (2015). “There’s a pain app for that”: review of patient-targeted smartphone applications for pain management. Clin J Pain.

[51] Jurcik D, Ross E, Sundaram A, Jamison R (2016). (181) utilization of a smartphone app for chronic pain care: does this improve outcome?. J Pain.

[52] Portelli P, Eldred C (2016). A quality review of smartphone applications for the management of pain. Br J Pain.

[53] Suso-Ribera C, Castilla D, Zaragozá I, Ribera-Canudas M, Botella C, García-Palacios A (2018). Validity, Reliability, Feasibility, and usefulness of pain monitor: a multidimensional smartphone app for daily monitoring of adults with Heterogenous chronic pain. Clin J Pain.

[54] Jamison RN, Jurcik DC, Edwards RR, Huang C, Ross EL (2017). A pilot comparison of a smartphone app with or without 2-way messaging among chronic pain patients: who benefits from a pain app?. Clin J Pain.

[55] Hayden JA, Dunn KM, van der Windt DA, Shaw WS (2010). What is the prognosis of back pain?. Best Pract Res Clin Rheumatol.

[56] DIGITALGOV: User Acceptance Testing Versus Usability Testing…What’s the Dif? https://digital.gov/2014/10/06/user-acceptance-testing-versus-usability-testing-whats-the-dif/. Accessed 2nd Oct 2018. 2018.

[57] Pechenkina E, Laurence D, Oates G, Eldridge D, Hunter D (2017). Using a gamified mobile app to increase student engagement, retention and academic achievement. Int J Educ Technol High Educ.

[58] Goren A, Mould-Quevedo J, daCosta DiBonaventura M (2014). Prevalence of pain reporting and associated Health outcomes across emerging markets and developed countries. Pain Med.

[59] Keele Pain Recorder: iTunes. 2017. https://itunes.apple.com/gb/app/keele-pain-recorder/id1287410877?mt=8. Accessed 25th Oct 2017.

[60] Keele Pain Recorder: Play. 2017. https://play.google.com/store/apps/details?id=com.keele.painrecorder2&hl=en_GB. Accessed 9th October 2018.

