# Supplementary material for: Development and validation of a pain monitoring app for patients with musculoskeletal conditions (The Keele pain recorder feasibility study)
Source: BMC Med Inform Decis Mak. 2019 Jan 25;19:24. doi: 10.1186/s12911-019-0741-z (PMC6347830; doi:10.1186/s12911-019-0741-z)
Supplement: Supplementary file 1 — Keele Pain Recorder Screenshots. Screenshots of data capture points, help page, and frequently asked question (FAQ) page in the Keele Pain Recorder App. (DOCX 408 kb) [file 12911_2019_741_MOESM1_ESM.docx]

Additional file 1 - Screenshots of data capture points, help page, and frequently asked question (FAQ) page in the Keele Pain Recorder App


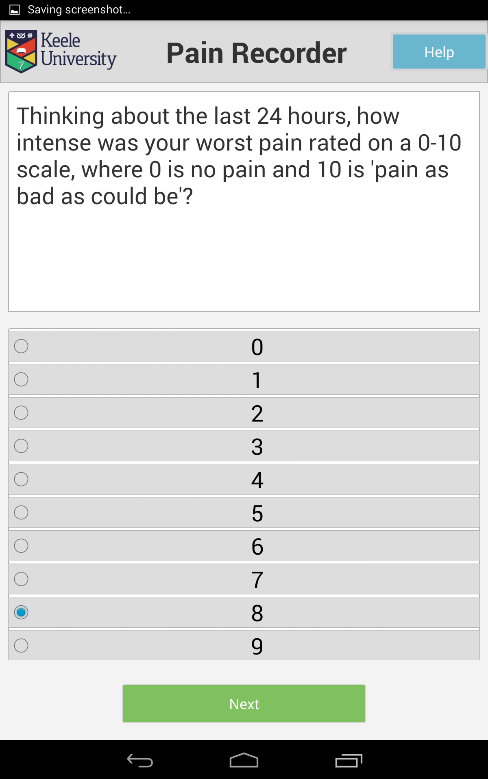


Screenshot 1 – Numerical rating scale for pain intensity


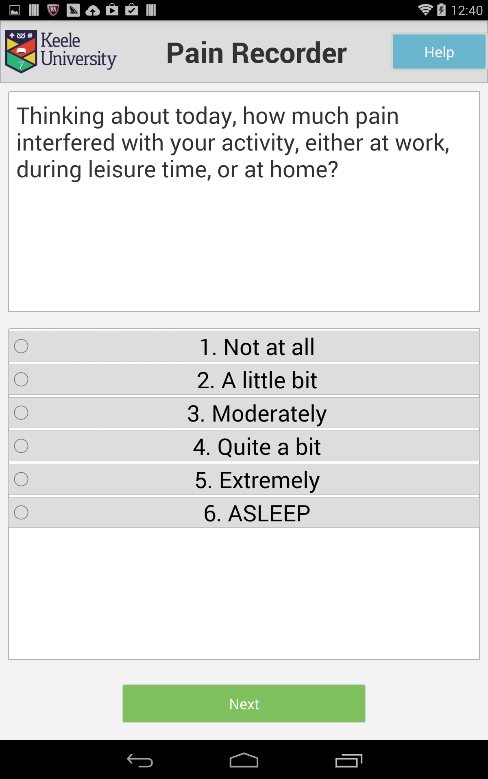


Screenshot 2 – Pain interference scale


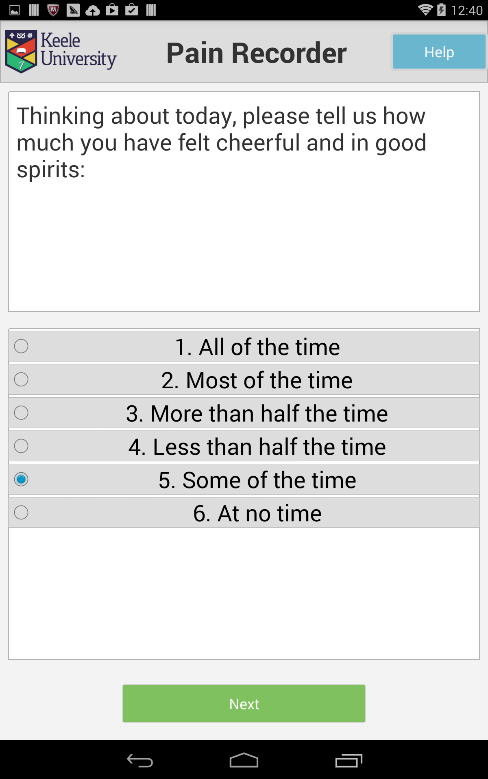


Screenshot 3 – WHO- wellbeing index


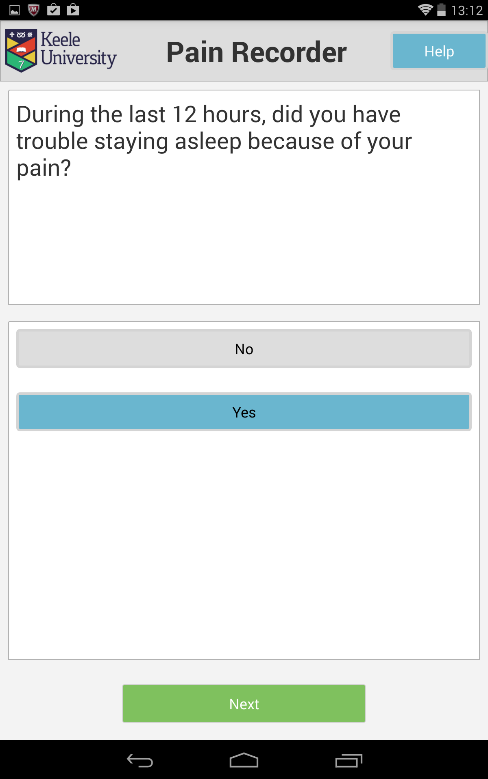


Screenshot 4 – Sleep disturbance


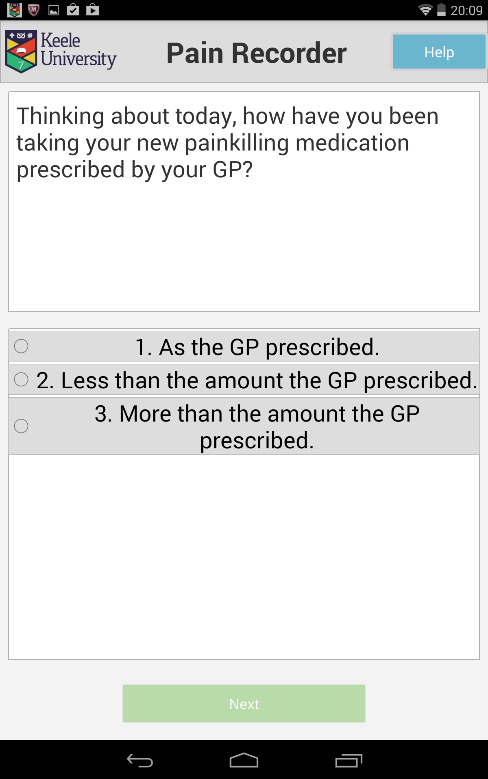


Screenshot 5 – Analgesic use


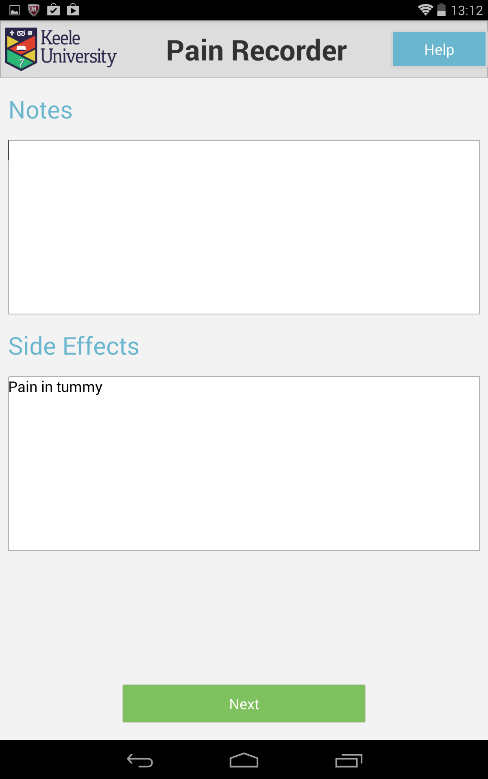


Screenshot 6 – Side effect recording


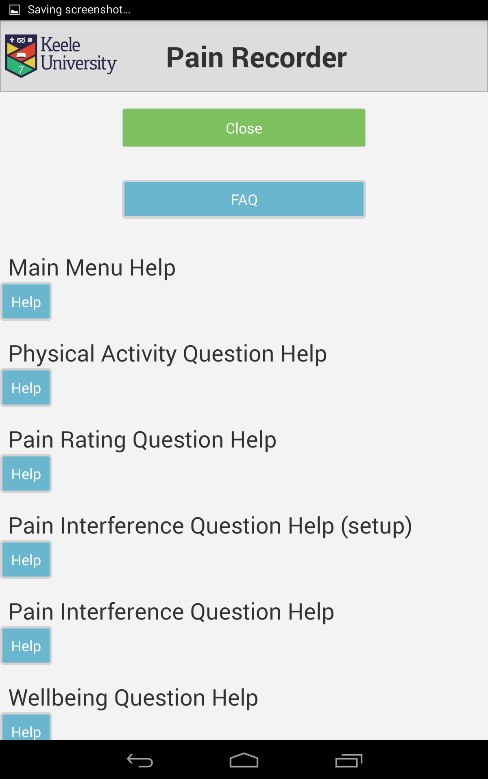


Screenshot 7 – Help page links


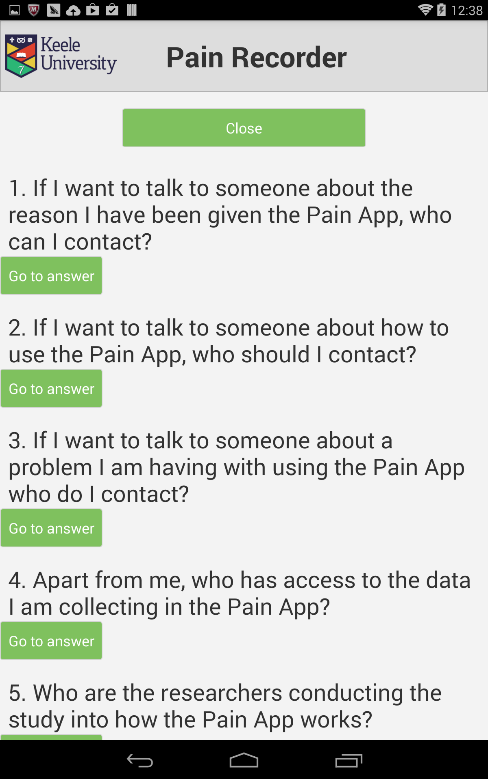


Screenshot 8 – Frequently asked questions (FAQ) page and links
